# CLINICIAN PERSPECTIVES ON PRE-VISIT AGENDA-SETTING FOR PERSONS WITH DEMENTIA AND THEIR CARE PARTNERS

**DOI:** 10.1093/geroni/igad104.3229

**Published:** 2023-12-21

**Authors:** Jessica Cassidy, Catherine DesRoches, Isabel Hurwitz, Jennifer Wolff, Hillary Lum

**Affiliations:** University of Texas at Arlington, Arlington, Texas, United States; Beth Israel Deaconess Medical Center, Boston, Massachusetts, United States; Beth Israel Deaconess Medical Center, Boston, Massachusetts, United States; Johns Hopkins University, Baltimore, Maryland, United States; Univerisity of Colorado School of Medicine, Aurora, Colorado, United States

## Abstract

Family care partners often assist persons with dementia in managing their health care, including through healthcare system patient portals. Thus, gaps in communication with family care partners can lead to unmet care needs. OurNotes is an agenda setting intervention included within pre-visit questionnaires that is sent through the patient portal. The intervention aims to encourage patients to share concerns before the visit to enhance communication between patients and clinicians. The objective of this study was to understand clinician perspectives on use of OurNotes in dementia care, including engaging care partners and suggestions for improving the intervention. Semi-structured interviews were conducted with 20 clinicians from two health systems with experience using OurNotes and caring for patients with dementia. Using rapid assessment methods, a codebook was developed and used in a team-based, thematic analysis. Participants were majority White, female, between 30-39 years, worked in primary care, and physicians. Clinicians identified: 1) Benefits of pre-visit preparation that enhanced clinician knowledge of the patient and/or care partner agenda; 2) Improved delivery of family-centered dementia care through greater care partner involvement; 3) Challenges of balancing patient and care partner concerns during clinical visits; and 4) Suggestions for focused efforts on improving care partners’ accessibility and use of OurNotes (and the patient portal). We conclude that optimizing use of OurNotes by care partners may support effective agenda setting by patients, care partners, and clinicians, and ultimately improve dementia care.

